# Surface Color Variations of Ground Beef Packaged Using Enhanced, Recycle Ready, or Standard Barrier Vacuum Films

**DOI:** 10.3390/foods11020162

**Published:** 2022-01-08

**Authors:** Tristan M. Reyes, Hunter R. Smith, Madison P. Wagoner, Barney S. Wilborn, Tom Bonner, Terry D. Brandebourg, Soren P. Rodning, Jason T. Sawyer

**Affiliations:** 1 Department of Animal Sciences, Auburn University, Auburn, AL 36849, USA; tzr0039@auburn.edu (T.M.R.); hzs0101@auburn.edu (H.R.S.); mpw0035@auburn.edu (M.P.W.); wilbobs@auburn.edu (B.S.W.); tdb0006@auburn.edu (T.D.B.); rodnisp@auburn.edu (S.P.R.); 2 Winpak Ltd., 100 Saulteaux Crescent, Winnipeg, MB R3J 3T3, Canada; tom.bonner@winpak.com

**Keywords:** ground beef, instrumental color, shelf life, vacuum packaging

## Abstract

With current meat industry efforts focused on improving environmental influencers, adopting sustainable packaging materials may be an easier transition to addressing the sustainability demands of the meat consumer. With the growing popularity of vacuum-packaged meat products, the current study evaluated instrumental surface color on fresh ground beef using vacuum packaging films, recycle-ready film (RRF), standard barrier (STB) and enhanced barrier (ENB). Ground beef packaged using ENB barrier film was lighter (L*), redder (a*) and more vivid (chroma) than all other packaging treatments during the simulated display period (*p* < 0.05). By day 12 of the simulated retail display, the ground beef surface color became lighter (L*), more yellow (b*), less red (a*), less vivid (chroma) and contained greater forms of calculated metmyoglobin, oxymyoglobin (*p* < 0.05). The current results suggest that barrier properties of vacuum packaging film for ground beef are pivotal for extending the surface color during fresh shelf-life conditions.

## 1. Introduction

When consumers purchase fresh meat, a primary attribute influencing the consumer’s purchasing decision is meat color [1]. As meat is stored in refrigerated conditions following harvest, fabrication, or portioning, surface color variations are dependent on the chemical state of myoglobin. With the presence of oxygen, the chemical state of myoglobin can appear as a bright, cherry-red color, often correlated to freshness by the retail consumer [2] at the time of selection and purchase. Vacuum packaging is often not the preferred packaging platform of choice within the retail consumer market setting by meat industry retailers due to surface color variations that are presented under vacuum. It is widely known that beef packaged in a vacuum platform displays a purple-red color identified as deoxy-myoglobin. Before vacuum packaging, fresh beef appears as a bright, cherry-red (oxy-myoglobin) color due to surface exposure to oxygen. When packaging fresh meat with limited exposure to oxygen (one to two days post packaging), the oxygen-bound myoglobin is converted to deoxymyoglobin [3] as a result of a reduction in partial pressure of the package. The reduction in partial pressure within a vacuum package of fresh meat can result in a shift in color form of myoglobin from a bright red oxygenated color to a purplish red deoxygenated appearance. It has been reported that 74% of consumers utilized color as an important attribute influencing their purchasing intent of meat [4]. Moreover, when half of the consumers are informed on vacuum-packaged beef and its purple-red color, consumers are more likely to purchase vacuum-packaged beef over non-barrier polyvinyl chloride (PVC) packaged beef [4]. Because of the unique fresh characteristics, ground beef is a product that often has a reduced shelf life because of the manufacturing process creating a greater surface area for oxidative and degradative reactions [5].

Vacuum packaging has been a resource often used for decades when packaging meat to achieve extended storage periods and meet the fresh and frozen product shelf-life expectations throughout the meat industry. To achieve vacuum packaging platforms, the use of plastic film is inevitable, and it is required to protect consumer foods from contaminates while maintaining product shelf life and potentially reducing food waste [6]. Efforts in previous years have mainly focused on shelf life and surface color of fresh beef. Countless studies have evaluated fresh meat under retail conditions using packaging platforms such as the following: polyvinyl chloride (PVC) with an overwrapped foam tray [7,8,9] appearance; modified atmosphere packaging (MAP) with various gasses [10,11,12]; newer efforts using PVC overwrapping with mother bag packaging [13,14,15] that combines MAP gases and atmospheric transmission of PVC. In recent years, efforts have suggested the use of high-barrier, multi-layer, biodegradable food packaging could be beneficial as a replacement to current multi-layered film packaging, which lacks the ability to be recycled [16].

It is evident with the growing trends in use of vacuum packaging, the investigation of recyclable materials and various films used within the vacuum packaging platform is necessary. Thermoforming vacuum packaging is constructed by the forming of multi-layered films with heat, pressure, and forming duration [17]. Moreover, many combinations of materials exist in the formation of these multi-layer films; some of these materials include amorphous polyethylene terephthalate (A-PET), polyolefins (PO), ethylene vinyl alcohol (EVOH), polyvinylidene di-chloride (PVdC), and Nylon [17,18,19,20]. Although films typically comprised of PO and EVOH have been constructed with the intent to be recycled downstream of the consumer [17], with the development of recyclable films, a challenge remains present due to limitations in the recycling process of flexible multi-layered films. A difficulty with recycling multi-layered films is the delamination of the individual layers within the constructed film which is currently not economically viable [17,18]. Nonetheless, efforts have been focused on identifying a viable option to recycle multi-layered films which include the technique of compatibilization, which can be done to produce a blended material [18]. In addition to compatibilization, the process of chemical recycling has been investigated as an option to recycle multi-layer packaging [17]. As technology within the packaging landscape of fresh meat evolves, the investigation of viable options in recycling multi-layer films is still essential to identifying the shelf-life performance of packaging films that could serve as a recycle ready option for the meat industry. Therefore, the objectives of this study were to evaluate the instrumental changes in surface color of fresh ground beef packaged in enhanced (ENB), standard (STB), and recycle-ready (RRF) vacuum packaging films and stored under simulated retail conditions.

## 2. Materials and Methods

### 2.1. Raw Materials

Coarse ground beef (80:20; lean:fat) packaged in 4.5 kg chubs (DuraChub, WINPAK, Winnipeg, MB, Canada) with an oxygen transmission rate of 0.9 cc/sg. m/24 h) was purchased from a commercial meat processing facility. Fresh, never-frozen chubs were transported under refrigerated conditions 1.5 °C ± 0.5 °C in the absence of light to the Auburn University Lambert Powell meat laboratory. Coarse ground beef was stored in the absence of light at 2.0 °C ± 1.0 °C for 48 h prior to grinding and packaging. At the time of grinding, coarse ground beef (36 kg) was allocated randomly to 1 of 3 treatments (*n* = 12 kg/treatment) and ground once using a commercial meat grinder (Model 4346, Hobart Corporation, Troy, OH, USA) through a 3.18 mm plate (SPECO 400, Schiller Park, IL, USA). After grinding, ground beef was portioned into 454 g bricks using a vacuum stuffer (Model-VF608plus, Handtmann, Biberach, Germany).

### 2.2. Packaging

Ground beef bricks (*n* = 5/treatment/rep) were placed into a commercial packaging film (WINPAK, Winnipeg, MB, Canada) consisting of an enhanced barrier (175 µm nylon, enhancedEVOH, and polyethylene: ENB), standard barrier (175 µm nylon, EVOH, and Polyethylene: STB), and recycle ready film (175 µm polyolefins and EVOH: RRF). The non-forming film used for all packages was comprised of (75 µm polyester, EVOH, and polyethylene). The oxygen transmission rates (OTR) for each packaging treatment were as follows: ENB (0.2 cc/sq. m/24 h); STB (0.4 cc/sq. m/24 h); RRF (0.5 cc/sq. m/24 h). However, the moisture vapor transmission rates for each treatment were as follows: ENB (3.3 g/sq. m/24 h); STB (3.3 g/sq. m/24 h); RRF (2.8 g/sq. m/24 h). Packages of ground beef bricks were sealed using a Variovac Optimus (OL0924, Variovac, Zarrentin am Schaalsee, Germany). After packaging, ground beef brick packages were individually identified and placed into dark storage at 2.2 °C ± 0.5 °C for 120 h.

### 2.3. Retail Display

Packaged ground beef was stored in the absence of light at 2.2 °C ± 0.5 °C for 120 h to simulate logistic conditions from manufacturer to retailer. Following dark storage, ground beef packages were placed into a three-tiered, lighted display case Turbo Air (Model 60DXB-N, Turbo Air Inc., Long Beach, CA, USA) operating at 3.0 °C ± 1.5 °C with three 25 min defrost cycles occurring each day. Shelf-life timeline for measuring surface color began (Day 0) at the time of displaying ground beef packages under constant lighting for simulated retail conditions. The lighting within the retail case consisted of cool LED strips (TOM-600-12-v4-3, Philips Xitanium 40W-75W, Korea) with a lighting intensity of 2297 lux (ILT10C, International Light Technologies, Peabody, MA, USA) on each shelf. Ground beef packages were randomly dispersed throughout the display case shelves and rotated daily to simulated consumer packaging shifting that occurs at the retail counter.

### 2.4. Instrumental Color

Throughout the 15-day simulated retail display period, instrumental surface color was measured on packages of ground beef (*n* = 75) with a HunterLab MiniScan EZ colorimeter, Model 45/0 LAV (Hunter Associates Laboratory Inc., Reston, WV, USA). Prior to surface color readings, the colorimeter was standardized using a black and white tile covered with the packaging films to confirm instrument accuracy. Surface color readings were captured each day of the simulated display period at 17:00. Instrumental color values were determined from the mean of three readings on the surface of each ground beef through the intact package using illuminant A, with an aperture of 31.8 mm, and a 10° observer to measure lightness (L*), redness (a*) and yellowness (b*) of each ground beef package [21]. In addition, hue angle was calculated as follows: tan^−^^1^ (b*/a*), with a greater value indicative of the surface color shifting from red to yellow. Chroma (C*) was calculated as: √a*^2^ + b*^2^ where a larger value indicates a more vivid color. Lastly, reflectance values within the spectral range 400 to 700 nm were used to capture the surface color changes from red to brown by calculating the reflectance ratio of 630 nm:580 nm and the relative percentages of deoxymyoglobin (DMB = {[1.395 − ({A572 − A700}**/**{A525 − A700})]} × 100), metmyoglobin (MMB = {2.375 × [1 − ({A473 − A700}**/**{A525 − A700})]} × 100), and oxymyoglobin (OMB = DMB − MMB) according to [22] Meat Color Measurement Guidelines.

### 2.5. Statistical Analysis

All data were analyzed as a completely randomized design using the ground beef package as the experimental unit with 25 replications of each treatment. The ANOVA was generated using the GLIMMIX model procedure of SAS (version 9.2; SAS Inst. Inc. Cary, NC, USA) using day of simulated retail display as a repeated measure, with packaging, day, and packaging × day interaction as the fixed effects. Least squares means were generated, and, when significant (*p* < 0.05) *F*-values were observed, least squares means were separated using pair-wise *t*-test (PDIFF option).

## 3. Results and Discussion

There were no (*p* > 0.05) interactive effects for packaging film × day throughout the simulated retail display period on surface color values of vacuum-packaged ground beef. Ground beef displayed using ENB barrier packaging film was lighter (*p* < 0.05) L* than ground beef packaged using STB or RRF (Table 1). Moreover, ground beef packages became lighter (*p* < 0.05) as the duration of storage time increased (Table 2). Similar results for surface lightness (L*) have been noted when using various packaging methods such as vacuum-packaged, overwrapping, or the addition of gasses within the package of fresh ground meat [23]. Additionally, fresh meat under lighted display in a limited oxygen package has been reported to impact the formation of oxymyoglobin formation [24]. Changes that occurred to the surface color lightness (L*) are likely a function of deoxy-myoglobin formation that occurred during the simulated display period. Furthermore, declining changes in lightness are similar with previous studies reporting vacuum-packaged ground beef became darker over a 20-day simulated display period following temperature abuse [23]. Ground beef packages were redder and more vivid (*p* < 0.05) when displayed using ENB packaging film, whereas RRF packages were more yellow (b*) and had a greater (*p* < 0.05) hue angle (Table 1). Nonetheless, redness and vividness declined (*p* < 0.05) as storage duration in refrigerated display increased (Table 2). A decrease in redness (a*) values suggest fresh meat products may be less accepted by consumers, due to the meat product presenting a darker red surface color. Previous studies have evaluated the influence of storage period and packaging method on beef steaks (*M. longissimus dorsi*) [25]. Similar results in vacuum-packaged steaks indicate a* values may decline throughout the storage period [25]. Moreover, similar results for vacuum-packaged beef loins have been reported to the current study, indicating chroma values (surface color vividness) will decline as the duration of display increases [26].

Instrumental spectral reflectance data from 400 to 700 nm was used to calculate relative values for the red to brown ratio (630/580 nm), metmyoglobin (MMB), deoxymyoglobin (DMB), and oxymyoglobin (OMB) of ground beef surface color changes. The red to brown ratio for ground beef packaged using ENB barrier packaging film was greater (*p* < 0.05) than ground beef packaged in STB or RRF films (Table 1). Additionally, throughout the duration of the retail display period, the red to brown (630/580 nm) values declined (*p* < 0.05), resulting in a shift from a redder to browner surface color (Table 2). As noted in previous research, red to brown values tend to decline regardless of packaging method [27]. It is plausible the shift in calculated red to brown values is a function of greater metmyoglobin formation over the course of the extended display period. Moreover, relative calculated values of oxymyoglobin captured through instrumental measurements indicated vacuum packages of ground beef in RRF film were greater (*p* < 0.05) than packages of ground beef in STB or ENB packaging films, respectively (Table 1). Surprisingly, calculated values of OMB increased (*p* < 0.05) in vacuum-packaged ground beef as the day of simulated display increased (Table 2). Interestingly, ground beef packaged using RRF films resulted in greater (*p* < 0.05) calculated relative values for MMB and OMB than ground beef packaged in STB or ENB packaging films (Table 1). However, as the time of display in days increased, calculated MMb increased (*p* < 0.05) and DMB values declined (Table 2). Changes recorded in calculated relative values of DMB, MMB, and OMB may be attributed to the oxygen transmission rates of the packaging films. In addition, it has been reported that the ratio of myoglobin forms in fresh meat can be influenced by the available oxygen, oxygen consumption rate, autoxidation of myoglobin or the reducing ability of metmyoglobin [28,29]. Current results for calculated myoglobin forms are similar to previous reports when evaluating vacuum-packaged fresh meats [30]. Furthermore, vacuum packaging of fresh meat can result in residual quantities of oxygen that may influence the autoxidation of DMB and MMB during extended storage periods [31]. However, it is plausible vacuum packaging resulted in a greater regeneration of NADH which has been reported to delay discoloration of fresh meats [32]. Nonetheless, it is widely known that a greater percentage of surface MMB greatly influences consumer purchasing intent at the retail counter. Regardless of the shelf-life duration for meat products, surface color and spoilage organism may contribute to the changes associated with a shift in surface color from DMB to MMB [3]. Therefore, the addition of continued research evaluating visual surface color of vacuum-packaged fresh meats is necessary.

## 4. Conclusions

Evaluation of vacuum packaging films for ground beef platforms indicated that ENB film provided a significant packaging solution for sustaining the fresh surface color of ground beef during a simulated retail display. When using ENB packaging films a reduction in surface color variation across the 15-day simulated retail display was noted when compared to STP and RRF packaging films. It is plausible the lack of color stability in ground beef surface color with RRF packaging film may have been impacted by the EVOH layer that exists within the layers of the packaging film. Furthermore, research evaluating RRF film is needed to identify the feasibility of vacuum packaging fresh meat, extension of shelf-life, reduction in lipid oxidation and visual surface color changes that may occur with a packaging film intended to be recycled after consumer use.

## Figures and Tables

**Table 1:** Influence of packaging film on color values of vacuum-packaged ground beef during a simulated retail display.

	TRT ^1^			
	ENB	STB	RRF	SEM *
Lightness (L*) ^2^	48.94 ^a^	48.38 ^b^	48.11 ^c^	0.044
Redness (a*) ^2^	21.02 ^a^	18.39 ^b^	16.92 ^c^	0.096
Yellowness (b*) ^2^	13.47 ^c^	14.04 ^b^	14.64 ^a^	0.028
C* ^3^	25.02 ^a^	23.26 ^b^	22.55 ^c^	0.067
Hue (°) ^4^	32.90 ^c^	37.93 ^b^	41.58 ^a^	0.188
RTB ^5^	2.61 ^a^	2.17 ^b^	1.91 ^c^	0.016
MMB (%) ^6^	25.22 ^c^	33.66 ^b^	39.83 ^a^	0.321
DMB (%) ^6^	66.73 ^a^	50.29 ^b^	40.68 ^c^	0.525
OMB (%) ^6^	8.05 ^c^	16.05 ^b^	19.49 ^a^	0.218

^1^ Packaging treatments are defined as follows: enhanced EVOH + polyethylene (ENB); nylon + EVOH + polyethylene (STB); and polyolefins + EVOH (RRF). ^2^ L* Values are a measure of darkness to lightness (larger value indicates a lighter color); a* values are a measure of redness (larger value indicates a redder color); and b* values are a measure of yellowness (larger value indicates a more yellow color). ^3^ C* (Chroma) is a measure of total color (larger number indicates a more vivid color). ^4^ Hue (°) angle represents the change in color from the true red axis (larger number indicates a greater shift from red to yellow). ^5^ RTB is the reflectance ratio of 630 nm ÷ 580 nm and represents a change in the color of red to brown (larger value indicates a redder color). ^6^ Calculated percentages of oxymyoglobin (OMB), deoxymyoglobin (DMB), and metmyoglobin (MMB) using relative spectral values. ^a–c^ Mean values within a row lacking common superscripts differ (*p* < 0.05). * SEM, Standard error of the mean.

**Table 2:** Influence of retail display (d) on color values of vacuum-packaged ground beef.

	Instrumental Value								
	L* ^1^	a* ^1^	b* ^1^	C* ^2^	Hue (°) ^3^	RTB ^4^	MMB (%) ^5^	DMB (%) ^5^	OMB (%) ^5^
Day 0	47.36 ^g^	22.05 ^a^	13.35 ^h,i^	25.86 ^a^	31.39 ^g^	3.05 ^a^	20.81 ^h^	69.85 ^a^	9.34 ^i,j^
Day 1	48.03 ^f^	21.66 ^a,b^	13.20 ^i^	25.42 ^b^	31.53 ^g^	2.88 ^b^	22.06 ^h^	69.18 ^a^	8.76 ^j^
Day 2	48.27 ^e,f^	21.37 ^b,c^	13.39 ^h^	25.28 ^b,c^	32.26 ^g^	2.78 ^b^	22.75 ^h^	67.05 ^a^	10.21 ^g,h,i^
Day 3	48.57 ^d^	20.80 ^c,d^	13.71 ^f,g^	24.99 ^c,d^	33.63 ^f^	2.59 ^c^	25.78 ^g^	63.31 ^b^	10.91 ^g,h^
Day 4	48.60 ^c,d^	20.61 ^d^	13.68 ^g^	24.82 ^d^	33.82 ^f^	2.53 ^c,d^	26.96 ^f,g^	62.72 ^b^	10.32 ^g,h,i^
Day 5	48.98 ^a^	20.45 ^d^	13.88 ^f^	24.80 ^d^	34.47 ^f^	2.44 ^d^	28.33 ^f^	61.77 ^b^	9.90 ^h,i,j^
Day 6	48.96 ^a,b^	19.78 ^e^	14.07 ^e^	24.37 ^e^	35.84 ^e^	2.31 ^e^	30.65 ^e^	57.79 ^c^	11.56 ^g^
Day 7	48.98 ^a,b^	18.62 ^f^	14.26 ^d^	23.58 ^f^	37.97 ^d^	2.10 ^f^	34.78 ^d^	51.36 ^d^	13.85 ^f^
Day 8	48.95 ^a,b^	18.04 ^f^	14.35 ^c,d^	23.18 ^f,g^	39.07 ^d^	2.02 ^f^	36.24 ^d^	48.82 ^d^	14.93 ^f^
Day 9	48.84 ^a,b,c^	17.42 ^g^	14.50 ^a,b,c^	22.80 ^g^	40.35 ^c^	1.91 ^g^	38.36 ^c^	44.55 ^e^	17.09 ^e^
Day 10	48.71 ^b,c,d^	16.81 ^h^	14.55 ^a,b^	22.37 ^h^	41.47 ^b,c^	1.85 ^g,h^	39.69 ^b,c^	41.90 ^e,f^	18.41 ^d,e^
Day 11	48.46 ^d,e^	16.42 ^h,i^	14.63 ^a^	22.13 ^h^	42.29 ^a,b^	1.80 ^h,i^	40.87 ^b^	39.84 ^f,g^	19.29 ^c,d^
Day 12	48.09 ^f^	15.54 ^j^	14.34 ^c,d^	21.26 ^j^	43.13 ^a^	1.67 ^j^	43.50 ^a^	34.43 ^h^	22.07 ^a^
Day 13	48.11 ^f^	16.04 ^i,j^	14.45 ^b,c^	21.69 ^i^	42.48 ^a,b^	1.74 ^i,j^	41.59 ^a,b^	37.88 ^g^	20.53 ^b,c^
Day 14	48.27 ^e,f^	16.03 ^i,j^	14.37 ^c,d^	21.64 ^i,j^	42.39 ^a,b^	1.75 ^i,j^	41.14 ^b^	38.03 ^g^	20.83 ^a,b^
SEM *	0.097	0.214	0.063	0.149	0.419	0.035	0.718	1.174	0.488

^1^ L* Values are a measure of darkness to lightness (larger value indicates a lighter color); a* values are a measure of redness (larger value indicates a redder color); and b* values are a measure of yellowness (larger value indicates a more yellow color). ^2^ C* (Chroma) is a measure of total color where a larger number indicates a more vivid color. ^3^ Hue (°) angle) represents the change from the true red axis where a larger number indicates a greater shift from red to yellow. ^4^ RTB Calculated as 630 nm reflectance/580 nm reflectance which represents a change in the color of red to brown (larger value indicates a redder color). ^5^ Calculated percentages of oxymyoglobin (OMB), deoxymyoglobin (DMB), and metmyoglobin (MMB) using relative spectral values. ^a^^–^^j^ Mean values within a column lacking common superscripts differ (*p* < 0.05). * SEM, Standard error of the mean.

## Data Availability

Not Applicable.

## References

[1] Montgomery J.L., Parrish F.C., Olson D.G., Dickson J.S., Niebuhr S. (2003). Storage and packaging effects on sensory and color characteristics of ground beef. Meat Sci..

[2] Gupta J., Bower C.G., Cavender G.A., Sullivan G.A. (2018). Effectiveness of different myoglobin states to minimize high pressure induced discoloration in raw ground beef. LWT.

[3] Claus J.R. (2007). Color Changes in Cooked Beef. Centennial, CO: Research and Knowledge Management-National Cattlemen’s Beef Association. https://www.beefresearch.org/resources/product-quality/fact-sheets/color-changes-in-cooked-beef.

[4] Lynch N.M., Kastner C.L., Kropf D.H. (1986). Consumer Acceptance of Vacuum Packaged Ground Beef as Influenced by Product Color and Educational Materials. J. Food Sci..

[5] Uboldi E., Zanoletti M., Franzetti L., Limbo S. (2015). Master bag low-oxygen packaging system: Quality evolution of ground beef patties during storage, blooming and display presentation. Food Packag. Shelf Life.

[6] Barlow C.Y., Morgan D.C. (2013). Polymer film packaging for food: An environmental assessment. Resour. Conserv. Recycl..

[7] Martin J.N., Brooks J.C., Brooks T.A., Legako J.F., Starkey J.D., Jackson S.P., Miller M.F. (2013). Storage length, storage temperature, and lean formulation influence the shelf-life and stability of traditionally packaged ground beef. Meat Sci..

[8] Petersen J.H., Togeskov P., Hallas J., Olsen M.B., Jørgensen B., Jakobsen M. (2004). Evaluation of retail fresh meat packagings covered with stretch films of plasticized PVC and non-PVC alternatives. Packag. Technol. Sci..

[9] Steele K.S., Weber M.J., Boyle E.A.E., Hunt M.C., Lobaton-Sulabo A.S., Cundith C., Hiebert Y.H., Abrolat K.A., Attey J.M., Clark S.D. (2016). Shelf life of fresh meat products under LED or fluorescent lighting. Meat Sci..

[10] Hunt M.C., Mancini R.A., Hachmeister K.A., Kropf D.H., Merriman M., DelDuca G., Milliken G. (2004). Carbon Monoxide in Modified Atmosphere Packaging Affects Color, Shelf Life, and Microorganisms of Beef Steaks and Ground Beef. J. Food Sci..

[11] Jayasingh P., Cornforth D.P., Carpenter C.E., Whittier D. (2001). Evaluation of carbon monoxide treatment in modified atmosphere packaging or vacuum packaging to increase color stability of fresh beef. Meat Sci..

[12] Jeong J.Y., Claus J.R. (2010). Color stability and reversion in carbon monoxide packaged ground beef. Meat Sci..

[13] Isdell E., Allen P., Doherty A.M., Butler F. (1999). Colour stability of six beef muscles stored in a modified atmosphere mother pack system with oxygen scavengers. Int. J. Food Sci. Technol..

[14] Limbo S., Uboldi E., Adobati A., Iametti S., Bonomi F., Mascheroni E., Santagostino S., Powers T.H., Franzetti L., Piergiovanni L. (2013). Shelf life of case-ready beef steaks (*Semitendinosus* muscle) stored in oxygen-depleted master bag system with oxygen scavengers and CO_2_/N_2_ modified atmosphere packaging. Meat Sci..

[15] Uboldi E., Lamperti M., Limbo S. (2013). Low O_2_ Master Bag for Beef Patties: Effects of Primary Package Permeability and Structure. Packag. Technol. Sci..

[16] Dilkes-Hoffman L.S., Lane J.L., Grant T., Pratt S., Lant P.A., Laycock B. (2018). Environmental impact of biodegradable food packaging when considering food waste. J. Clean. Prod..

[17] Pauer E., Tacker M., Gabriel V., Krauter V. (2020). Sustainability of flexible multilayer packaging: Environmental impacts and recyclability of packaging for Bacon in block. Clean. Environ. Syst..

[18] Kaiser K., Schmid M., Schlummer M. (2017). Recycling of polymer-based multilayer packaging: A Review. Recycling.

[19] Dixon J. (2011). Packaging Materials: 9. Multilayer Packaging for Food and Beverages.

[20] Cenci-Goga B.T., Iulietto M.F., Sechi P., Borgogni E., Karama M., Grispoldi L. (2020). New Trends in Meat Packaging. Microbiol. Res..

[21] Commission Internationale de l’ Eclairage (1978). Recommendations on Uniform Color Spaces—Color Difference Equations, Psychometric Color Terms.

[22] American Meat Science Association (2012). Meat Color Measurement Guidelines.

[23] Rogers H.B., Brooks J.C., Martin J.N., Tittor A., Miller M.F., Brashears M.M. (2014). The impact of packaging system and temperature abuse on the shelf life characteristics of ground beef. Meat Sci..

[24] Greene B.E., In-May H., Zipser M.Y.W. (1971). Retardation of oxidative color changes in ground beef. J. Food Sci..

[25] Bağdatli A., Kayaardi S. (2014). Influence of storage period and packaging methods on quality attributes of fresh beef steaks. CyTA-J. Food.

[26] Strydom P.E., Hope-Jones M. (2014). Evaluation of three vacuum packaging methods for retail beef loin cuts. Meat Sci..

[27] Stivarius M.R., Pohlman F.W., McElyea K.S., Apple J.K. (2002). Microbial, instrumental color and sensory color and odor characteristics of ground beef produced from beef trimmings treated with ozone or chlorine dioxide. Meat Sci..

[28] Ledward D.A., Johnston D.E., Knight M.K. (1992). Colour of raw and cooked meat. The Chemistry of Muscle-Based Foods.

[29] Mancini R.A., Hunt M.C. (2005). Current research in meat color. Meat Sci..

[30] Li X., Lindahl G., Zamaratskaia G., Lundström K. (2012). Influence of vacuum skin packaging on color stability of beef *longissimus lumborum* compared with vacuum and high-oxygen modified atmosphere packaging. Meat Sci..

[31] George P., Stratmann C.J. (1952). The oxidation of myoglobin to metmyoglobin by oxygen 2. The relation between the first order rate constant and the partial pressure of oxygen. Biochem. J..

[32] Ramanathan R., Mancini R.A., Joseph P., Yin S., Tatiyaborworntham N., Petersson K.H., Sun Q., Konda M.R. (2011). Effects of lactate on ground lamb colour stability and mitochondria-mediated metmyoglobin reduction. J. Food Chem..

